# Editorial: Advancements in ischemic retinopathies: unraveling molecular mechanisms and innovative therapeutic avenues

**DOI:** 10.3389/fmed.2026.1853495

**Published:** 2026-05-13

**Authors:** Dario Rusciano, Snježana Kaštelan, Pamela M. Martin, Ravirajsinh N. Jadeja

**Affiliations:** 1 Neurovisual Science Technology (NEST), Catania, Italy; 2 Department of Ophthalmology, Clinical Hospital Dubrava, School of Medicine, University of Zagreb, Zagreb, Croatia; 3 School of Graduate Studies, Meharry Medical College, Nashville, TN, United States

**Keywords:** acute glaucoma, anti-VEGF, diabetic retinopathy, ischemic retinopathies, photocoagulation, retinal vascular occlusion, retinopathy of prematurity

Ischemic retinopathies remain among the most challenging disorders in ophthalmology to understand and manage (1). Although impaired perfusion is often the initiating event, the resulting damage rarely remains confined to the retinal vasculature. Metabolic stress increases, inflammatory pathways are activated, oxidative injury accumulates, the blood-retinal barrier weakens, and neural tissue begins to suffer (1). By the time these changes become clinically evident, the disease process is often already well-established. For that reason, progress in this field depends not only on better treatments but also on a more integrated understanding of how vascular, metabolic, inflammatory, and neurodegenerative mechanisms interact across different retinal diseases (2, 3). The studies collected in this Research Topic reflect that shift particularly well. Taken together, they suggest that ischemic retinopathies are best understood not as isolated vascular disorders but as complex diseases of the retinal neurovascular unit.

One of the most valuable aspects of this Research Topic is the way it connects molecular mechanisms with clinically meaningful questions. This is especially clear in the papers focused on diabetic retinal disease. Gao et al. examine endothelial dysfunction under hyperglycemic stress and show that ATG16L1 is upregulated in retinal capillary endothelial cells exposed to high glucose, with corresponding increases in migration and invasion. The fact that silencing ATG16L1 attenuates these responses is important, as it suggests that diabetic retinal vascular damage is not merely a passive consequence of metabolic overload but may also depend on active intracellular programs that promote pathological remodeling. In this sense, their study opens a useful mechanistic window onto early diabetic microvascular injury. Liu et al. then extend this line of thinking from intracellular signaling to the ocular microenvironment by identifying increased carbonic anhydrase-1 (CA-1) in the aqueous humor of eyes with diabetic macular edema and linking it to inflammatory mediators and vascular dysfunction. Read together, these two studies do more than add separate pieces of information: they help bridge cell-level mechanisms and fluid-phase biomarkers, reinforcing the idea that diabetic retinal disease evolves through multiple, interacting layers of pathology.

That broader biological framework is developed further in the mini-review by Lester et al., which focuses on GPR109A as a possible link among inflammation, oxidative stress, and retinal barrier dysfunction across several ischemic retinopathies. The value of this contribution lies not only in the receptor itself, but in the perspective it offers. Conditions such as diabetic retinopathy, hypertensive retinal injury, and retinopathy of prematurity are often discussed as separate entities, yet many of the pathways that drive retinal damage appear to overlap. By highlighting a metabolite-sensing pathway that may operate across disease boundaries, this review helps move the discussion away from rigid diagnostic categories and toward a more mechanism-based view of ischemic retinal injury.

Another strong theme running through this Research Topic is the search for biomarkers that can define disease activity earlier and with greater precision. Zhu et al. show that GDF15 is elevated in retinal artery occlusion and may improve diagnostic discrimination when combined with metabolic and inflammatory parameters. In disorders where the therapeutic window can be narrow, this kind of marker is especially appealing. It is not difficult to imagine how such a signal, if validated further, could contribute not only to diagnosis but also to risk stratification. Liu et al., from a different but complementary angle, support the same biomarker-oriented movement through local aqueous humor profiling in diabetic macular edema. Together, these studies suggest that the future of ischemic retinal disease assessment may depend increasingly on biologically informed markers that bring molecular insight closer to clinical decision-making.

The practical clinical dimension of this Research Topic is equally noteworthy. Barazi et al. describe a case in which paracentral acute middle maculopathy (PAMM) served as a retinal sign of carotid artery dissection, reminding us that retinal ischemic findings may carry significance well beyond the eye itself. This case is compelling because it highlights the retina as a sensitive window into systemic vascular disease. A similar emphasis on clinically useful recognition appears in the work of Li et al., who validated the G-ROP criteria in a Chinese cohort and showed that the model successfully identified all infants requiring treatment while reducing unnecessary screening examinations. Although these two contributions differ substantially in design and clinical context, they point in the same direction: better retinal care depends not only on treatment, but on accurate and timely identification of the patients who most need attention.

The therapeutic contributions in this Research Topic also deserve to be read in relation to one another rather than in isolation. Rusciano et al. revisit prostaglandin E1 and make a thoughtful case for reconsidering its vasoactive, anti-inflammatory, and potentially neuroprotective effects in ischemic retinal disease. What makes this review especially useful is its balance: it does not overstate the evidence, yet it invites the field to rethink older pharmacologic approaches in light of present-day pathophysiological knowledge. Carnevali et al., in turn, examine the long-term efficacy and safety of dexamethasone intravitreal implants in retinal vein occlusion. This study confirms the continued clinical value of corticosteroid-based therapy while also acknowledging the well-known limitations of this strategy, particularly intraocular pressure elevation and cataract progression. Taken together, these reviews point to an important conclusion: future treatment of ischemic retinopathies will likely depend less on any single intervention and more on combining vascular, anti-inflammatory, metabolic, and neuroprotective strategies in a rational, patient-specific way.

**Figure 1:**
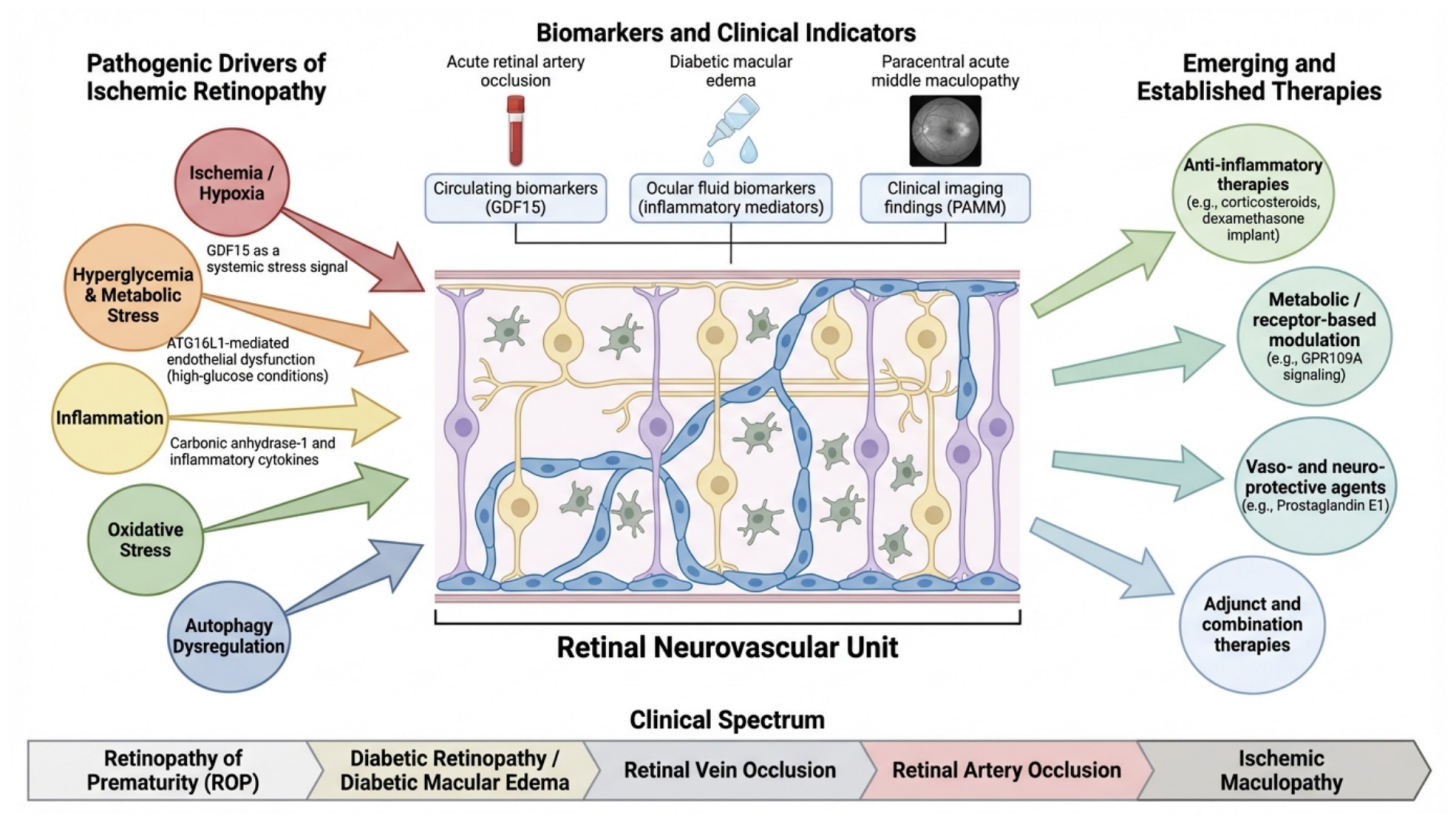
Pathogenic mechanisms, clinical indicators, and therapeutic avenues across the spectrum of ischemic retinopathies. This schematic summarizes major pathogenic drivers of ischemic retinopathies, including hypoxia/metabolic stress, inflammation, oxidative stress, and autophagy dysregulation, and their collective effects on the retinal neurovascular unit. It also highlights circulating, ocular-fluid, and imaging-based biomarkers and indicators, including GDF15, inflammatory mediators, diabetic macular edema, acute retinal artery occlusion, and paracentral acute middle maculopathy (PAMM). Emerging and established therapeutic approaches are shown, including anti-inflammatory agents, metabolic and receptor-based modulation, vaso- and neuroprotective therapies, and adjunctive or combination treatments. The lower panel presents the clinical continuum of ischemic retinopathies, including retinopathy of prematurity, diabetic retinopathy, diabetic macular edema, retinal vein occlusion, retinal artery occlusion, and ischemic maculopathy. This shared view emphasizes common pathophysiological mechanisms and may support the development of therapies targeting fundamental disease processes. This illustration was created using FigureLabs.ai.

This Research Topic offers a unified view of an increasingly mechanistic, clinical, and integrated field. The studies explain the development of retinal injury, propose refined diagnostic methods, show how retinal findings improve screening and reveal systemic diseases, and contextualize therapies within a translational framework. Its main contribution is to move beyond a fragmented view of ischemic retinopathies toward a shared understanding that links mechanisms, phenotypes, and therapies (Figure 1). The message is timely: ischemic retinopathies are complex neurovascular diseases influenced by metabolic, inflammatory, and degenerative processes. This approach promotes better biomarkers, smarter screening, early detection, and integrated therapies. We hope it benefits scientists and clinicians, inspiring progress toward safer, effective treatments for ischemic retinal diseases.
